# Improving Bone Health by Optimizing the Anabolic Action of Wnt Inhibitor Multitargeting

**DOI:** 10.1002/jbm4.10462

**Published:** 2021-05-06

**Authors:** Roy B Choi, Whitney A Bullock, April M Hoggatt, Gabriela G Loots, Damian C Genetos, Alexander G Robling

**Affiliations:** ^1^ Department of Anatomy, Cell Biology & Physiology Indiana University School of Medicine Indianapolis IN USA; ^2^ Biology and Biotechnology Division Lawrence Livermore National Laboratory Livermore CA USA; ^3^ Molecular Cell Biology Unit, School of Natural Sciences University of California Merced Merced CA USA; ^4^ Department of Anatomy, Physiology, and Cell Biology University of California–Davis School of Veterinary Medicine Davis CA USA; ^5^ Richard L. Roudebush Veterans Affairs Medical Center Indianapolis IN USA; ^6^ Department of Biomedical Engineering Indiana University–Purdue University at Indianapolis Indianapolis IN USA; ^7^ Indiana Center for Musculoskeletal Health Indianapolis IN USA

**Keywords:** BONE ANABOLISM, OSTEOPOROSIS, SCLEROSTIN Dkk1, Wnt

## Abstract

Sclerostin antibody (romosozumab) was recently approved for clinical use in the United States to treat osteoporosis. We and others have explored Wnt‐based combination therapy to disproportionately improve the anabolic effects of sclerostin inhibition, including cotreatment with sclerostin antibody (Scl‐mAb) and Dkk1 antibody (Dkk1‐mAb). To determine the optimal ratio of Scl‐mAb and Dkk1‐mAb for producing maximal anabolic action, the proportion of Scl‐mAb and Dkk1‐mAb were systematically varied while holding the total antibody dose constant. A 3:1 mixture of Scl‐mAb to Dkk1‐mAb produced two to three times as much cancellous bone mass as an equivalent dose of Scl‐mAb alone. Further, a 75% reduction in the dose of the 3:1 mixture was equally efficacious to a full dose of Scl‐mAb in the distal femur metaphysis. The Scl‐mAb/Dkk1‐mAb combination approach was highly efficacious in the cancellous bone mass, but the cortical compartment was much more subtly affected. The osteoanabolic effects of Wnt pathway targeting can be made more efficient if multiple antagonists are simultaneously targeted. © 2021 The Authors. *JBMR Plus* published by Wiley Periodicals LLC. on behalf of American Society for Bone and Mineral Research.

## Introduction

The past three decades of osteoporosis drug development have witnessed Food and Drug Administration (FDA) approval of five different classes of antiresorptives, including hormones (eg, calcitonin), oral bisphosphonates (e.g., alendronate), SERMs (e.g., raloxifene), i.v. bisphosphonates (e.g., zoledronic acid), and antibodies (e.g., denosumab). Currently, more than 10 different pharmaceuticals are available for antiresorptive therapy to reduce fracture susceptibility. Yet in the same period, only two classes of bone anabolic agents (PTHR1 agonists and a Wnt inhibitor antagonist) have made it to the US market, one of which was approved only in the last year. The most recently approved bone‐forming therapeutic is a sclerostin antibody, romosozumab, which increases Wnt signaling in bone cells and promotes bone formation.

Sclerostin targeting is highly effective in increasing bone mass,^(^ ^1^ ^)^ which begs the question of whether there are other potent, secreted inhibitors of Wnt signaling that are amenable to targeting to produce osteoanabolic action. However, targeting some of the other Wnt inhibitors has yielded disappointing results. For example, Dkk1 antibody (Dkk1‐mAb) fails to generate a significant osteoanabolic response in adult rats^(^ ^2^ ^)^ and mice.^(^ ^3^ ^)^ However, Dkk1‐mAb is highly osteoanabolic in Sost^−/−^ mice^(^ ^3^ ^)^ and in WT mice cotreated with sclerostin antibody (Scl‐mAb),^(^ ^4^ ^)^ suggesting that sclerostin signaling suppresses the otherwise osteoanabolic effect of Dkk1 inhibition. Not only is Dkk1 inhibition anabolic in a Sost/sclerostin depleted environment, but a potentiated response to dual antibody treatment is generated, where the anabolic response is greater than either Scl‐mAb or Dkk1‐mAb alone, reaching significantly beyond additive effects.^(^ ^3^, ^4^ ^)^

One of the advantages of uncovering interaction among drug combinations is that they can facilitate a reduction in the total amount of drug required, which has manifold benefits, including (i) reducing cost simply by requiring less drug consumption^(^ ^5^ ^)^; (ii) reducing complications from drug volume, such as injection‐site reactions—a problem for Lilly's sclerostin antibody during phase 2^(^ ^6^ ^)^; and (iii) potentially reducing side effects of any one individual drug.^(^ ^7^ ^)^ Although combination therapy with Scl‐mAb/Dkk1‐mAb (at a 1:1 ratio) clearly potentiates the Scl‐mAb effect in bone, the optimal proportionality of the two reagents is not known. In this communication, we treated mice with various proportions of Scl‐mAb and Dkk1‐mAb, while keeping the dose of total antibody constant (25 mg/kg), to find the optimal ratio for anabolic action. Further, we serially reduced the total dose of optimized formulation administered to determine the total drug volume at which the optimal formulation performs similarly to the pure Scl‐mAb treatment (the current clinical option).

Our studies suggest that different combinations of agents targeting Wnt inhibitors can have potent potentiating effects while reducing required drug amounts, especially when delivered in the optimized proportions. Further, the bone envelope‐specific effects of targeting individual and/or multiple Wnt signaling components allows for selective modulation of cortical versus cancellous compartments for therapeutic benefit.

## Materials and Methods

### Experimental mice

Eight‐week‐old C57BL/6J female mice were purchased from Jackson Laboratory and acclimatized for 1 week prior to experimentation. All animal procedures were performed in accordance with relevant federal guidelines and conformed to the Guide for the Care and Use of Laboratory Animals (8th edition) as previously described.^(^ ^3^ ^)^ The animal facility at Indiana University is an Association for Assessment and Accreditation of Laboratory Animal Care International–accredited facility, and all mouse procedures were performed in accordance with the IACUC guidelines and approvals.

### Antibody injection

Details of the development of Dkk1‐ and sclerostin‐neutralizing antibodies have been reported elsewhere.^(^ ^18^, ^19^ ^)^ Briefly, the Dkk1 antibody (Dkk1‐mAb) is a neutralizing monoclonal rat mAb raised against mouse Dkk1. The sclerostin antibody (Scl‐mAb), which neutralizes mouse sclerostin, is a version of a mouse monoclonal antibody in which the amino acid sequence has been modified for use in rats. Antibodies were injected sc into mice beginning at 9 weeks of age at various doses (described in the experiments) twice per week. Vehicle treatment was the phosphate buffered saline, in which the antibodies were diluted. All mice were treated for 6 weeks.

### Dual‐energy x‐ray absorptiometry

Collection of repeated DXA measurements on live mice are described and validated elsewhere.^(^ ^1^ ^)^ Briefly, isoflurane‐anesthetized mice were scanned on a PIXImus II (GE Lunar) densitometer at the beginning of the treatment period (9 weeks of age) and again at the terminal timepoint (16 weeks of age). BMD was measured for the whole body (excluding the skull), lumbar spine (L3–L5), and right lower limb (distal to the acetabulum) using the Lunar ROI tools.

### Micro‐computed tomography

Formalin‐fixed femora and L5 lumbar vertebrae were scanned, reconstructed, and analyzed on a Scanco μCT‐35 as previously described.^(^ ^1^, ^20^ ^)^ We used10‐μm resolution, 50‐kV peak tube potential, and 151‐ms integration time. Standard parameters^(^ ^25^ ^)^ related to cancellous and cortical bone architecture were measured.

### Fluorochrome administration and bone quantitative histomorphometry

Mice were given 200‐μL injections of demeclocycline (40 mg/kg ip) at 9 weeks of age, calcein (12 mg/kg ip) at 14 weeks of age, and alizarin complexone (20 mg/kg ip) at 15 weeks of age to label mineralizing bone throughout the experimental period. After euthanization at 16 weeks of age, the femurs were processed for plastic‐embedded histomorphometry, and cut at midshaft for histological evaluation as previously described.^(^ ^24^ ^)^ Briefly, periosteal and endocortical mineralizing surface (MS/BS; %), mineral apposition rates (MAR; μm/d), and bone formation rates (BFR/BS; μm^3^/μm^2^/y) were calculated using the demeclocycline and calcein labels, measured over the entire periosteal and endocortical surfaces (not subregions) according to standard protocols.^(^ ^26^ ^)^ Alizarin labels were injected into mice (and thus they appear in the histology images shown), but were not used for measurements.

### Whole‐bone mechanical properties

Parameters related to whole‐bone strength were measured using three‐point bending tests as previously described.^(^ ^27^ ^)^ Briefly, each femur was loaded to failure in monotonic compression using three‐point bending platens. The lower span points were spaced 10 mm, and the upper point contacted the femoral diaphysis at midshaft. During each test, force and displacement were collected every 0.01 seconds. From the force/displacement curves, ultimate force and energy to failure were calculated using standard equations.^(^ ^28^ ^)^

### RNA isolation and quantitative polymerase chain reaction

Total RNA was isolated from tibia diaphyses (isolated cortical tubes with periosteum and marrow removed) using previously described cryo‐pulverization and Qiagen RNeasy kits.^(^ ^3^ ^)^ cDNAs were reverse transcribed from 250 ng of total RNA using high‐capacity cDNA reverse transcription kits (Applied Biosystems). Quantitative PCR was performed on an ABI Quantstudio Flex 7 (Applied Biosystems) using FastStart Universal Probe Master ROX mix (Roche). Sost and Dkk1 expression were calculated using the 2−ΔCt method, and normalized to transcripts for the housekeeper eukaryotic translation initiation factor3 (EIF3F). Sost (Mm00470479_m1) and Dkk1 (Mm00470479_m1) Taqman primer/probe mixes were purchased from ABI. EIF3F primers were customized by Roche Life Science Universal Probe Library (UPL) assay system: Left‐attcacctcacggtggaca Right‐agggacacccattaaagtgct.

### Serum C‐terminal telopeptide

Serum concentration of the resorption marker C‐terminal telopeptide (CtX) was measured by a commercially available ELISA (RatLaps; IDS Inc.) as previously described.^(^ ^1^, ^20^ ^)^ Briefly, blood samples were collected from O/N fasted 12‐week‐old mice at the retromandibular vein. Blood samples were permitted to clot at room temperature for 30 minutes, spun at 5000*g* to separate the serum, and frozen at −80°C. Thawed serum samples were assayed for CtX in triplicate according to the manufacturer's instructions.

### Statistical analysis

Statistical analyses were performed by one‐way ANOVA followed by post hoc Tukey‐HSD test using JMP (version 4.0; SAS Institute). Statistical significance was indicated by a *p* value of *p* < 0.05. All graphs are shown as box plots indicating the 25th to 75th interquartile range. The median value is denoted as a line within the box. Whiskers represent the data range.

## Results

### Combining sclerostin and Dkk1 antibody at a 3:1 ratio yields optimal bone gain, particularly in the cancellous compartment

**Fig 1:**
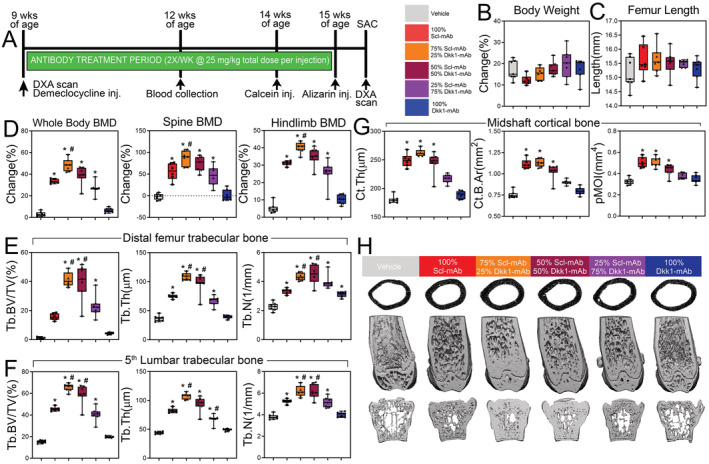
A 3:1 ratio of sclerostin to Dkk1 antibody produces maximal skeletal benefits in cancellous bone. (*A*) Experimental design and timeline, including, DXA scans, fluorochrome labels, blood draws, and antibody‐treatment duration. (*B*) Percent change in body mass of mice receiving 25 mg/kg of antibody at different relative proportions of Scl‐Ab and Dkk1‐mAb, calculated using beginning (9 week) and final (16 week) measurements. (*C*) Femur length at euthanization of all treatment groups at 16 weeks of age. (*D*) DXA‐derived changes in BMD, calculated using beginning (9 weeks) and final (16 weeks) measurements at three regions of interest: whole body (left panel), lumbar spine (middle panel), and entire right hindlimb distal to the acetabulum (right panel). (*E*) μCT‐derived trabecular bone volume fraction (Tb.BV/TV), thickness (Tb.Th), and number (Tb.N) in the distal femoral metaphysis of all treatment groups at 16 weeks of age. (*F*) μCT‐derived trabecular bone volume fraction (Tb.BV/TV), thickness (Tb.Th), and number (Tb.N) in the L5 lumbar vertebra of all treatment groups at 16 weeks of age. (*G*) μCT‐derived cortical bone thickness (Ct.Th), area (Ct.B.Ar), and polar moment of inertia (pMOI) at the femoral midshaft of all treatment groups at 16 weeks of age. (*H*) Representative μCT reconstructions of the femoral midshaft, distal femur, and L5 lumbar vertebra from each treatment group, revealing the potent effects of combination therapy (particularly the 3:1 formulation) in cancellous but not cortical bone. *p < 0.05 versus vehicle; #*p* < 0.05 versus Scl‐mAb alone; *n* = 6–7 mice/group.

We reported previously that combination therapy, involving sclerostin monoclonal antibody (Scl‐mAb) and Dkk1 monoclonal antibody (Dkk1‐mAb), generates more than double the amount of bone found for Scl‐mAb alone (and no response from Dkk1‐mAb alone). Those experiments used a 1:1 mixture of Scl‐mAb to Dkk1‐mAb, but there is no a priori reason to assume that a 1:1 mixture of the two inhibitors is the optimal ratio for maximizing potentiation. To tailor this therapeutic approach for maximal skeletal benefit, we explored the osteoanabolic outcome for different relative proportions of inhibitor. Nine‐week‐old female WT mice were treated for 6 weeks with various proportions of Scl‐mAb and Dkk1‐mAb (Fig. 1*A*), but the dose of total antibody (Scl‐mAb + Dkk1‐mAb) was kept constant at 25 mg/kg. Antibody treatment had no significant effect on body weight or femur length, suggesting that growth was not affected by the treatments (Fig. 1*B*,*C*). As expected, compared with vehicle control, Dkk1‐mAb alone (0:1) had no effect on BMD measured for whole body, spine, or lower limb, whereas Scl‐mAb alone (1:0) significantly increased BMD at all three ROIs (25%–58%; *p* < 0.01; Fig. 1*D*). All combinations of Scl‐mAb and Dkk1‐mAb antibody increased BMD beyond vehicle treatment, but only the 3:1 mixture improved BMD compared with Scl‐mAb alone (1:0). The 3:1 mixture increased BMD an additional 10% to 32% (*p* < 0.05) beyond Scl‐mAb alone, whereas the 1:1 and 1:3 formulations yielded gains in BMD that were not statistically different from Scl‐mAb alone.

μCT–derived cancellous bone properties in the distal femur and lumbar spine, including bone volume fraction (BV/TV), trabecular thickness (Tb.Th), number (Tb.N), and spacing (Tb.Sp) were significantly improved by Scl‐mAb alone, but were unchanged by Dkk1‐mAb alone, with the exception of femoral Tb.N and Tb.Sp (Fig. 1*E* & 1H, and Supplementary Fig. S1 *A*). The 3:1 antibody formulation significantly increased femoral BV/TV, Tb.N, and Tb.Th over Scl‐mAb treatment alone by twofold or more (*p* < 0.05). Likewise, the 1:1 formulation, but not the 1:3 formulation produced an increase in cancellous properties compared with Scl‐mAb alone. Cancellous properties in the lumbar vertebrae followed similar trends (Fig. 1*F* and Supplementary Fig. S2*B*).

### Combining sclerostin and Dkk1 antibody has marginal efficacy in improving cortical bone properties beyond Scl‐mAb alone

**Fig 2:**
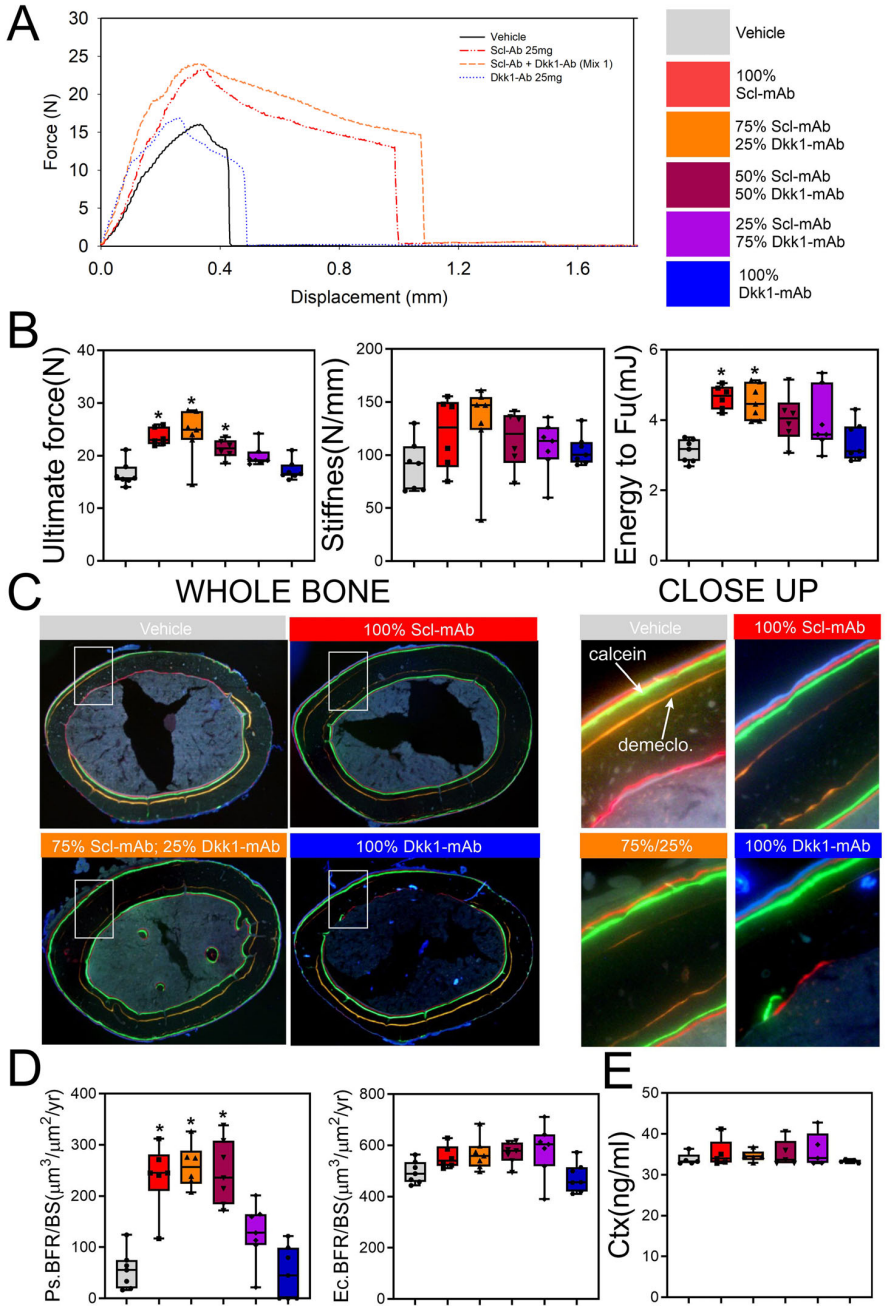
Cortical bone mechanical properties and formation indices are not improved by combination sclerostin/Dkk1 antibody therapy. (*A*) Representative force‐displacement curves from three‐point monotonic bending tests to failure conducted on whole femurs from 16‐week‐old mice treated with vehicle, Scl‐mAb alone, Dkk1‐mAb alone, or a 3:1 cocktail of Scl‐mAb/Dkk1‐mAb (remaining groups were omitted from panel for clarity). (*B*) Quantification of ultimate force (peak height of the curve in panel A), stiffness (slope of the linear portion of the curve in panel *A*), and energy absorbed (area under the curve in *A*). (*C*) Representative fluorochrome‐labeled midshaft femur histologic cross sections from mice treated as described for panel *A*. The ROI box in the whole‐bone panels is magnified in the right panels to visualize bone formation between the demeclocycline (orange) label and the calcein (green) label. Alizarin (red) labels were injected but not used for measurements. See Fig. 1A for labeling schedule. (*D*) Quantification of anabolic action on the periosteal (Ps) and endocortical (Ec) surfaces, measured using the demeclocycline and calcein labels (Alizarin labels were not used for measurements), and presented as the bone formation rate per unit bone surface (BFR/BS). Mineralizing surface and mineral apposition rates are given in Supplementary Fig. S2. (*E*) Quantification of serum concentration of C‐terminal telopeptide (CtX) from all treatment groups at 12 weeks of age. *p < 0.05 versus vehicle; *n* = 6–7 mice/group.

Although the trabecular bone compartment was dramatically improved by Scl‐mAb/Dkk1‐mAb combination therapy, particularly at the 3:1 and 1:1 ratios, no significant potentiation was detected in the cortical compartment among the mice treated with different ratios of Scl‐mAb:Dkk1‐mAb. None of the dual antibody formulations produced improvements in cortical bone area, thickness, or second moments beyond Scl‐mAb alone (Fig. 1 *G*). Moreover, the whole‐bone mechanical properties’ ultimate force, stiffness, and energy absorption (Fig. 2*A*,*B*), as well as histomorphometrically derived bone formation parameters (BFR, MAR, and MS/BS) at the femoral midshaft (Fig. 2*C*,*D* and Supplementary Fig. S2) were not improved by combination therapy at any ratio compared with Scl‐mAb alone. The resorption marker CtX was unaffected by any treatment (Fig. 2*E*).

**Fig 3:**
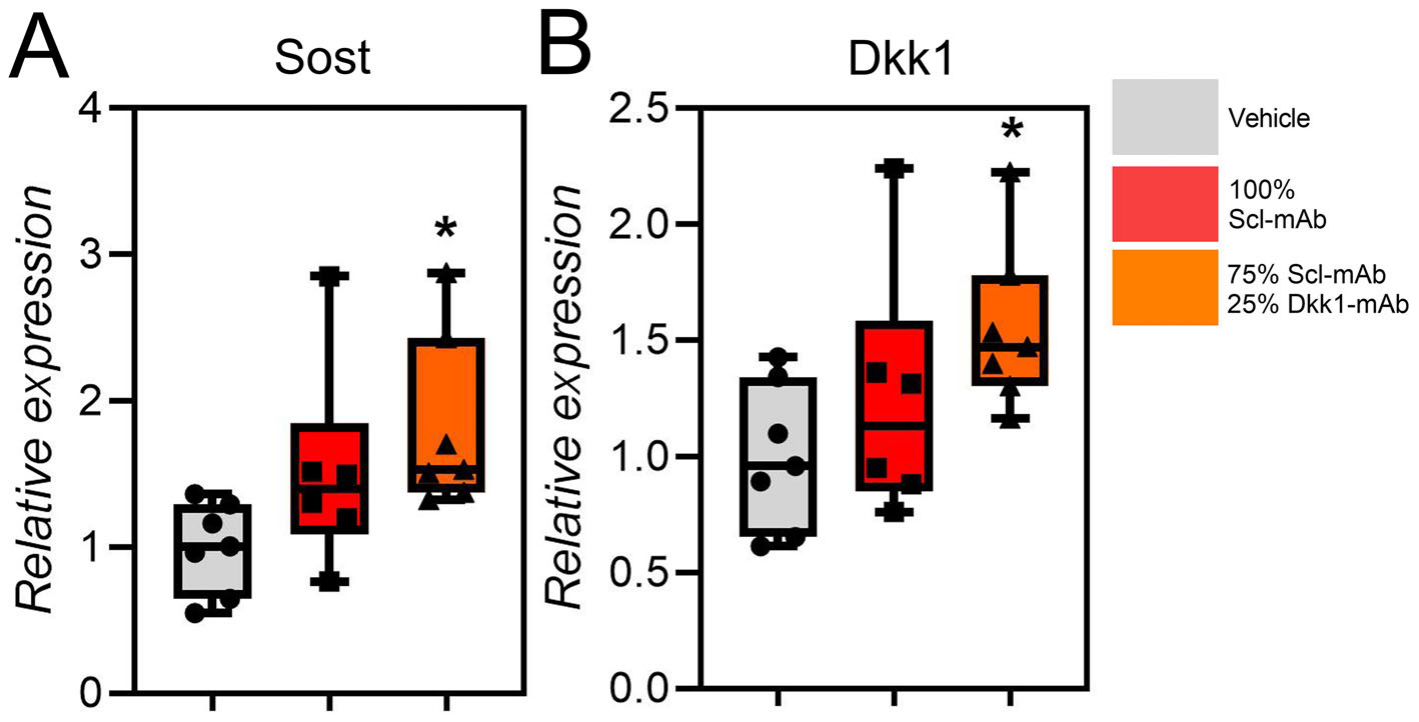
The 3:1 combination therapy increases expression of both Sost and Dkk1. (*A*) Relative gene expression levels of (*A*) Sost and (*B*) Dkk1 in vehicle, Scl‐mAb treated (25 mg/kg), and 3:1 combination therapy–treated (25 mg/kg) mice measured at 16 weeks of age. *p < 0.05 versus vehicle, *n* = 6–7 mice/group.

To assess compensation in inhibitor expression when neutralizing antibodies are injected, we measured the mRNA expression levels in cortical bone lysates from mice treated with vehicle, Scl‐mAb alone (25 mg/kg), and 25 mg/kg of 3:1 combination therapy. Compared with vehicle and Scl‐mAb treated groups, the 3:1 group showed significantly increased expression of both Sost and Dkk1, supporting the concept of self‐regulation previously proposed for Wnt inhibitors (Fig. 3*A,B*).^(^ ^8^ ^)^

### Scl‐mAb/Dkk1‐mAb administered at a 3:1 ratio is equally osteoanabolic to four times the dose of Scl‐mAb alone in cancellous bone

**Fig 4:**
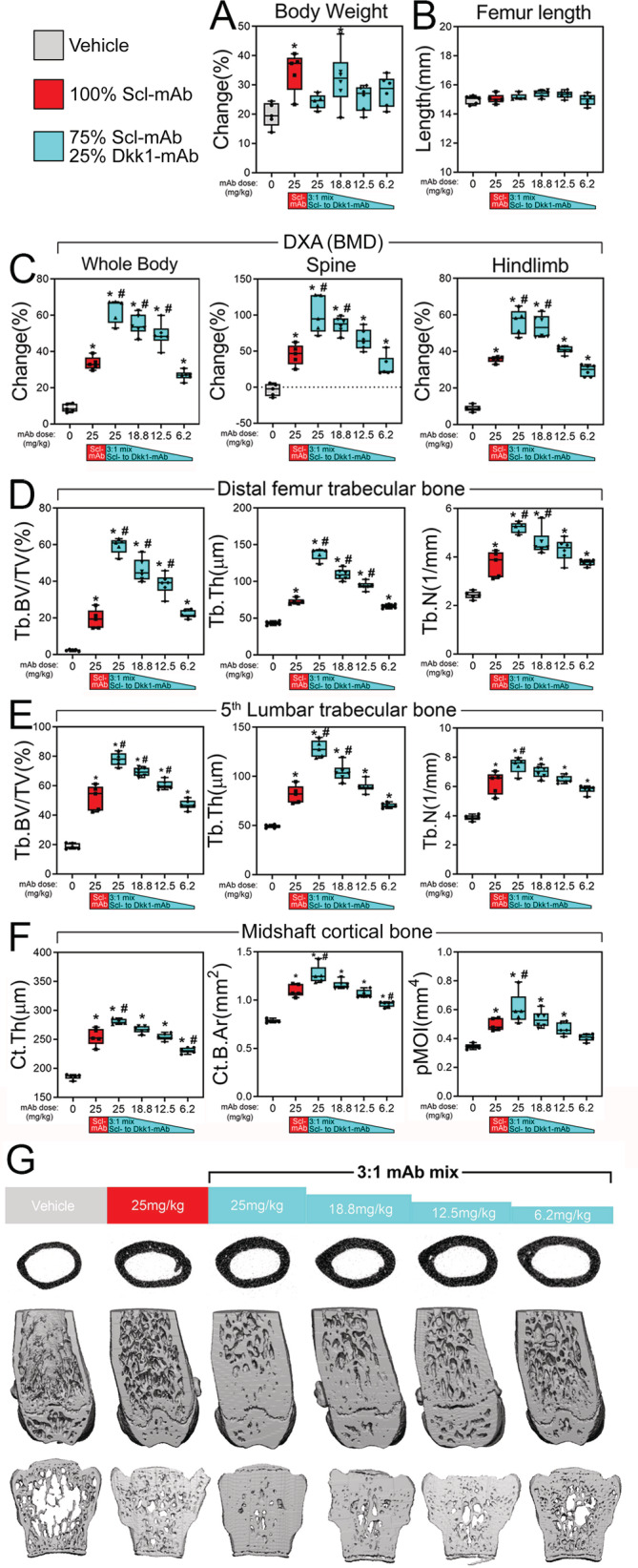
Very low total doses of combination therapy are as efficacious as high doses of Scl‐mAb. (*A*) Percent change in body mass of mice receiving different total dose of 3:1 Scl‐mAb/Dkk1‐mAb antibody mixture, calculated using beginning (9 week) and final (16 week) measurements. (*B*) Femur length at euthanization of all treatment groups at 16 weeks of age. (*C*) DXA‐derived changes in BMD, calculated using beginning (9 weeks) and final (16 weeks) measurements at three regions of interest: whole body (left panel), lumbar spine (middle panel), and entire right hindlimb distal to the acetabulum (right panel). (*D*) μCT‐derived trabecular bone volume fraction (Tb.BV/TV), thickness (Tb.Th), and number (Tb.N) in the distal femoral metaphysis of all treatment groups at 16 weeks of age. (*E*) μCT‐derived trabecular bone volume fraction (Tb.BV/TV), thickness (Tb.Th), and number (Tb.N) in the L5 lumbar vertebra in all treatment groups at 16 weeks of age. (*F*) μCT‐derived cortical bone thickness (Ct.Th), area (Ct.B.Ar), and polar moment of inertia (pMOI) at the femoral midshaft in all treatment groups at 16 weeks of age. (*G*) Representative μCT reconstructions of the femoral midshaft, distal femur, and L5 lumbar vertebra from each treatment group, revealing the potent effects of low‐dose combination therapy. *p < 0.05 versus vehicle; #*p* < 0.05 versus Scl‐mAb alone; *n* = 5–6 mice/group.

The previous experiments revealed that the Scl‐mAb/Dkk1‐mAb cocktail, given at a 3:1 ratio, resulted in two to three times as much cancellous bone as an equal dose of Scl‐mAb alone. Given the high potency of combination therapy, we next tested how small a dose of 3:1 Scl‐mAb/Dkk1‐mAb could be administered while still retaining equal potency to the Scl‐mAb–alone treatment (the current clinical analog). We tested this by treating 9‐week‐old female WT mice for 6 weeks with the 3:1 formulation at 25, 18.8, 12.5, or 6.2 mg/kg, and compared the effects to Scl‐mAb alone at 25 mg/kg. Dkk1‐mAb alone was excluded in this experiment because it is consistently similar to vehicle. Consistent with the previous experiment, antibody treatment had no significant effect on femur length, but body weight was significantly increased among two of the treated groups compared with vehicle control (Fig. 4*A*,*B*). DXA scans revealed that treatment with Scl‐mAb alone significantly increased BMD at all three ROIs—whole body, spine, and hindlimb—by 25% to 49% (*p* < 0.01), which was further increased by an equivalent 25 mg/kg dose of 3:1 antibody mixture (further 20%‐56% increase beyond Scl‐mAb alone; *p* < 0.01; Fig 4*C*). Reducing the total dose of the 3:1 formulation resulted in a stepwise reduction in BMD, but the 18.8 mg/kg dose of 3:1 still generated significantly more BMD than the 25 mg/kg Scl‐mAb–alone treatment at all three ROIs. The lowest 3:1 dose tested (6.2 mg/kg) increased BMD to the same degree as the 25 mg/kg Scl‐mAb–alone treatment.

In the distal femur metaphysis, the 25 mg/kg of 3:1 mixture generated nearly triple the amount of distal femur bone volume fraction (BV/TV) as an equal dose of Scl‐mAb alone (Fig. 4*D* and Supplementary Fig. S3*A*), a result that is consistent with the previous experiment. Stepwise reduction in the total dose of 3:1 Sost/Dkk1 antibody limited the efficacy of trabecular bone properties, but doses as low as 12.5 mg/kg were significantly more efficacious than the 25 mg/kg Scl‐mAb alone for BT/TV and Tb.Th. Moreover, the lowest 3:1 dose tested—6.2 mg/kg—was statistically similar to 25 mg/kg Scl‐mAb alone for all distal femur cancellous parameters, suggesting that a quarter dose of 3:1 is equally efficacious as a full dose of Scl‐mAb alone. Cancellous properties in the lumbar vertebrae followed similar trends (Fig. 4*E* and Supplementary Fig. S3*B*).

**Fig 5:**
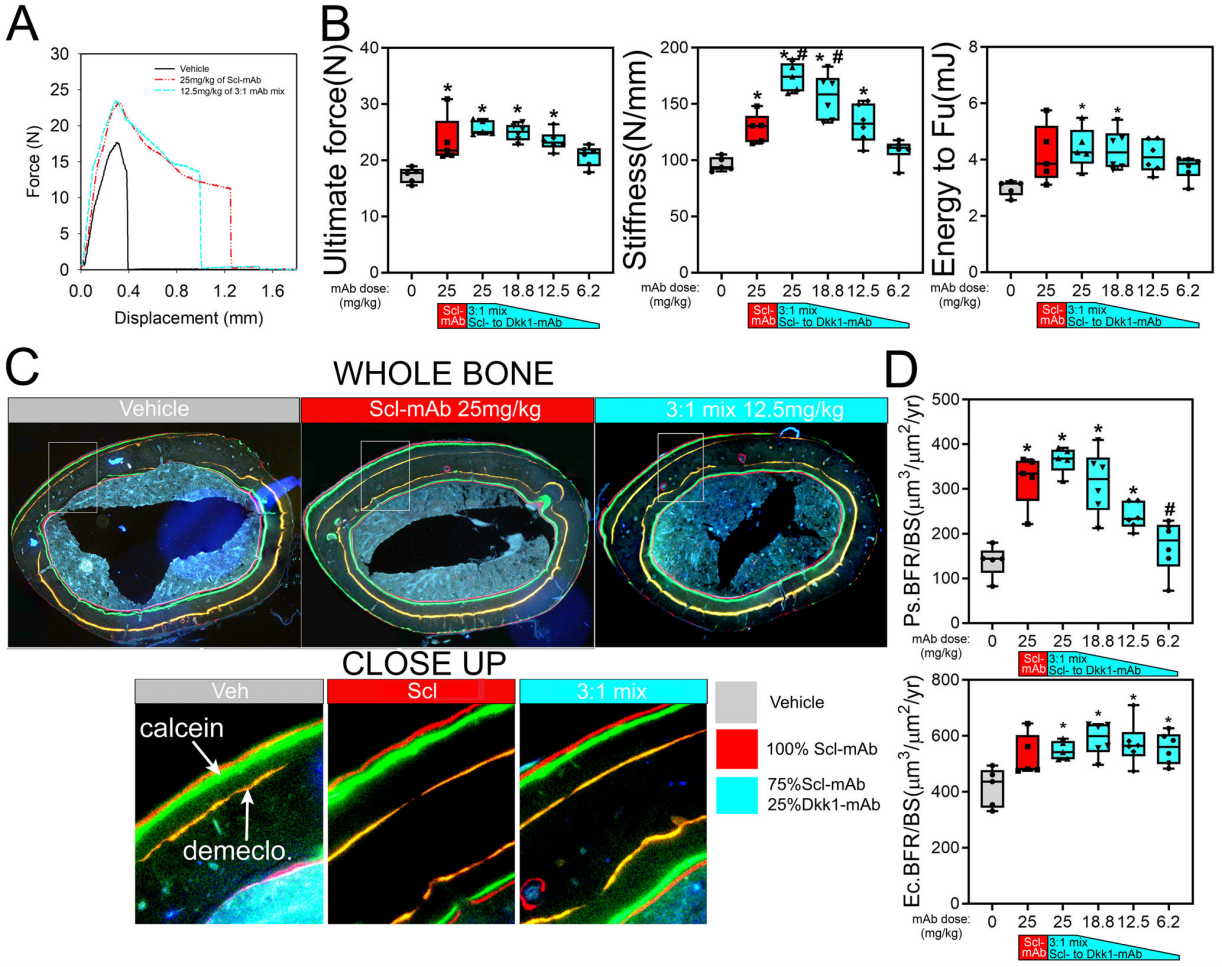
Very low doses of 3:1 combination therapy result in similar improvements in cortical bone mechanical properties and formation indices compared with high‐dose Scl‐mAb. (*A*) Representative force‐displacement curves from three‐point monotonic bending tests to failure conducted on whole femurs from 16‐week‐old mice treated with vehicle, 25 mg/kg Scl‐mAb alone, or 12.5 mg/kg of a 3:1 mixture of Scl‐mAb and Dkk1‐mAb (remaining dose groups were omitted from panel for clarity). (*B*) Quantification of ultimate force (peak height of the curve in panel a), stiffness (slope of the linear portion of the curve in panel a) and energy absorbed (area under the curve in a). (*C*) Representative fluorochrome‐labeled midshaft femur histologic cross sections from mice treated as described for panel a. The ROI box in the whole‐bone panels is magnified in the lower panels to visualize bone formation between the orange demeclocycline label and the red and green calcein and Alizarin labels, respectively. (*D*) Quantification of anabolic action on the periosteal (Ps) and endocortical (Ec) surfaces calculated as the bone formation rate per unit bone surface (BFR/BS). *p < 0.05 versus vehicle; #*p* < 0.05 versus Scl‐mAb alone; *n* = 5–6 mice/group.

We next examined cortical bone properties, using μCT, mechanical testing, and fluorochrome histomorphometry. Unlike the previous experiment, μCT‐derived cortical thickness, area, and polar moment were marginally but significantly improved by 3:1 antibody treatment at 25 mg/kg compared with the 25 mg/kg Scl‐mAb–alone treatment (11%‐22% increase; *p* < 0.05; Fig. 4*F*). Among the biomechanical (Fig. 5A & 5B) and histomorphometric (Fig. 5C & 5D) measurements, none of the 25 mg/kg 3:1 treatment groups yielded significant improvements over the 25 mg/kg Scl‐mAb‐alone group, with the exception of stiffness for the 25 mg/kg 3:1 group (Supplementary Fig. S4). All of the reduced 3:1 dose groups (18.8‐6.2 mg/kg) had statistically similar values to those found for the 25 mg/kg Scl‐mAb–alone group.

## Discussion

Our main goal in this study was to determine if the interaction of sclerostin/Dkk1 coinhibition could be optimized to improve the osteoanabolic outcome over Scl‐mAb alone, which is an FDA‐approved strategy for the treatment of osteoporosis. The premise for this inquiry was based on published observations that mice with bone‐selective codeletion of Sost and Dkk1 have disproportionately more bone mass than mice with either single mutation.^(^ ^3^ ^)^ Here, we found that a 3:1 ratio of Scl‐mAb to Dkk1‐mAb given at a total dose of 25 mg/kg resulted in two to three times as much trabecular bone as the same dose of Scl‐mAb alone. This is a remarkable finding given that Dkk1‐mAb alone has no consistent effect on bone properties.^(^ ^2^, ^3^, ^4^ ^)^ We further found that the 3:1 dose could be titrated down to 25% of the Scl‐mAb–alone dose while maintaining equal efficacy. These data suggest that a fraction of the injection volume and antibody content can be delivered while still maintaining high anabolic potency, which could be particularly useful if side effects of any one agent are of concern.

Consistent with known effects of Wnt inhibitor targeting in the mouse skeleton, the effects of combined antibody were driven by anabolic action, as the serum resorption marker CtX was not changed by either antibody alone or by combinations thereof. Previous animal studies using Scl‐mAb report inconsistent results regarding changes in resorption, where no change^(^ ^10^, ^11^, ^12^ ^)^ or significantly decreased^(^ ^13^, ^14^ ^)^ resorption have been found in response to antibody. Clinical studies have revealed early transient reductions in resorption markers among participants administered romosozumab.^(^ ^15^ ^)^ It is therefore possible that Scl‐mAb/Dkk1‐mAb treatment in patients might also have antiresorptive properties.

The Scl‐mAb/Dkk1‐mAb combination approach was highly efficacious in cancellous bone, but the cortical bone response to combination treatment was inconsistent. The first experiment showed no effect for any Scl‐mAb:Dkk1‐mAb formulation on cortical bone (ie, beyond the effects of Scl‐mAb alone). However, the second experiment indicated significant improvement in μCT‐derived properties among the 25 mg/kg 3:1 treatment compared with 25 mg/kg Scl‐mAb–alone treatment. Although these changes were not manifest in other assays of cortical bone gain (biomechanics, histomorphometry) and when present were much more subtle than those found for cancellous bone, we cannot rule out the possibility that the 3:1 formulation might have cortical bone effects as well. Further, the histomorphometric measurements exhibited a significant degree of variability. For example, the endocortical BFR/BS was not significantly increased by the 3:1 dose in the ratio (first) study, but it was in the total total dose (second) study, whereas the opposite case was observed for periosteal MAR. It is conceivable that antagonism of other combinations of Wnt inhibitors might have more potent effects in the cortical compartment, as manipulation of specific Wnt pathway components can selectively affect different compartments of the skeleton. For example, whereas sclerostin^(^ ^1^ ^)^ affects all bone compartments, sFrp4^(^ ^16^ ^)^ has opposing effects on cortical versus cancellous bone, and Wise^(^ ^9^ ^)^ and Wnt16^(^ ^17^ ^)^ selectively alter cortical bone in mice. As skeletal therapies become more sophisticated, it will be advantageous to selectively (or preferentially) target a particular bone surface or envelope. Further elucidation of mechanisms for other Wnt components highlights their potential utility for selectively modulating bone mass in surface‐ and compartment‐specific ways, whether used singly or in combination.

The experiments described have several limitations. First, though combination therapy can reduce off‐target effects by lowering doses, we do not know the off‐target effects of Dkk1‐mAb as well as for Scl‐mAb, which has been through rigorous clinical trials. Second, we used only female mice in this study. Significant sex effects have not been reported in the response to Scl‐mAb, we assume but cannot be sure that the potentiation effects are sex‐independent. Further, antibody potentiation in the presence of sex hormones Orchiectomy/Ovariectomy (ORX/OVX) or aging were not tested, which are perhaps more appropriate models for the patient population that would benefit from combined therapy. Finally, different antibodies will have different efficacies and pharmacokinetic profiles, so the optimal ratios found here might not apply to alternatively generated antibodies that target the same proteins.

In summary, the osteoanabolic effects of combination sclerostin‐ and Dkk1‐based inhibition can be tailored to provide maximal benefit to skeletal properties, while reducing the dose required to generate a robust anabolic response. The sclerostin/Dkk1 targeting approach is particularly beneficial for cancellous bone. As further advances are made in our understanding of the full spectrum of WNT inhibitors, other members of the secreted WNT inhibitory milieu might be identified that can provide additional skeletal benefits when combined.

## Author contributions

**Roy Choi:** Conceptualization; data curation; writing‐original draft.

## Conflict of Interest

All authors have declared that no conflicts of interest exist.

### Peer review

The peer review history for this article is available at https://publons.com/publon/10.1002/jbm4.10462.

## Supporting information


**Figure S1. (*A*)** μCT‐derived trabecular separation (Tb.Sp) and bone mineral content (Tb.BMC) in the distal femoral metaphysis among mice receiving 25 mg/kg of antibody at different relative proportions of Scl‐Ab and Dkk1‐mAb, at 16 weeks of age. **(*B*)** μCT‐derived trabecular separation (Tb.Sp) and bone mineral content (Tb.BMC) in the 5^th^ lumbar vertebra among all treatment groups, at 16 weeks of age. *p < 0.05 vs. vehicle; #*p* < 0.05 vs. Scl‐mAb alone; *n* = 6–7 mice/group.
**Figure S2.** Quantification of anabolic action on the **(*A*)** endocortical (Ec) and **(*B*)** periosteal (Ps) surfaces, measured using labels administered at the start (9 weeks) and near the end (14 weeks) of the antibody treatment period, and presented as the mineralizing surface per unit bone surface (MS/BS) and the mineral apposition rate (MAR). Mice received 25 mg/kg of antibody at different relative proportions of Scl‐Ab and Dkk1‐mAb, *p < 0.05 vs. vehicle; #*p* < 0.05 vs. Scl‐mAb alone; *n* = 6–7 mice/group.
**Figure S3.** μCT‐derived trabecular separation (Tb.Sp) and bone mineral content (Tb.BMC) in **(*A*)** the distal femoral metaphysis and **(*B*)** the 5^th^ lumbar vertebra among mice receiving different doses of 3:1 Scl‐mAb/Dkk1‐mAb, compared to vehicle control and Scl‐mAb alone (at 25 mg/kg). *p < 0.05 vs. vehicle; #*p* < 0.05 vs. Scl‐mAb alone; *n* = 5–6 mice/group.
**Figure S4.** Quantification of anabolic action on the **(*A*)** endocortical (Ec) and **(*B*)** periosteal (Ps) surfaces, measured using labels administered at the start (9 weeks) and near the end (14 weeks) of the antibody treatment period, and presented as the mineralizing surface per unit bone surface (MS/BS) and the mineral apposition rate (MAR). Mice received different doses of 3:1 Scl‐mAb/Dkk1‐mAb (blue rectangles), 25 mg/kg of Scl‐mAb alone (red rectangles), or vehicle control (gray rectangles). *p < 0.05 vs. vehicle; #*p* < 0.05 vs. Scl‐mAb alone; *n* = 5–6 mice/group.
