# Supplementary figures and images for: Improving Bone Health by Optimizing the Anabolic Action of Wnt Inhibitor Multitargeting
Source: JBMR Plus. 2021 May 6;5(5):e10462. doi: 10.1002/jbm4.10462 (PMC8101614; doi:10.1002/jbm4.10462)

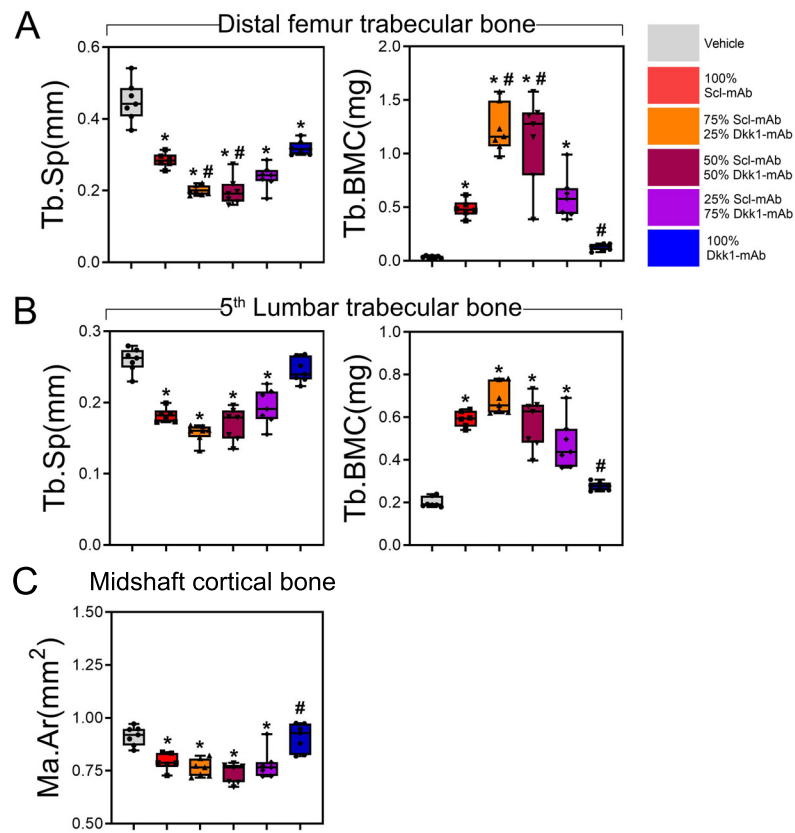

Figure S1

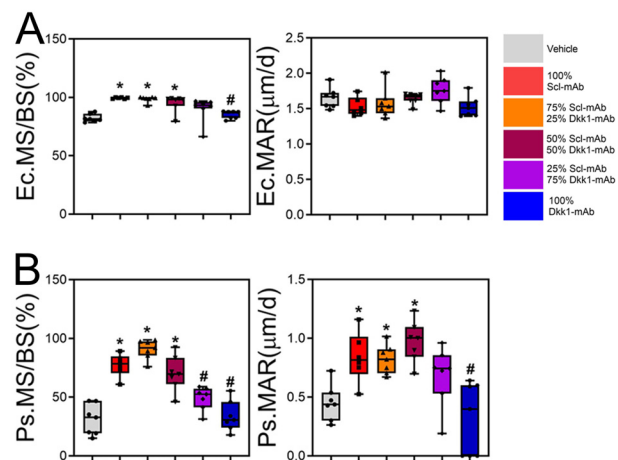

Figure S2

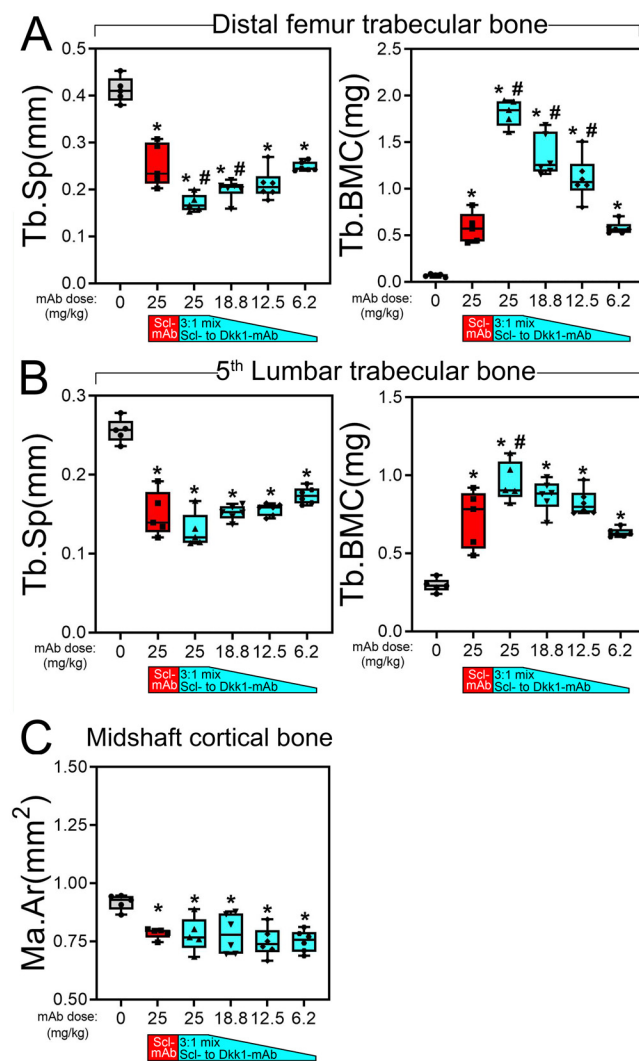

Figure S3

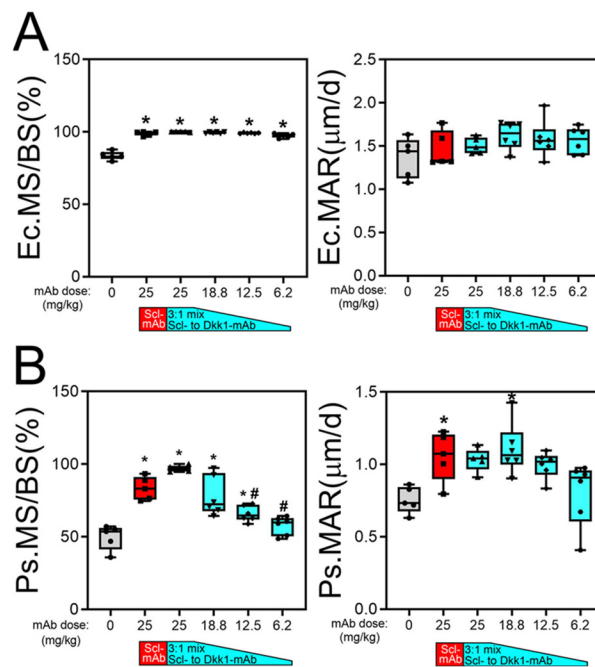

Figure S4

Supplement: Supplementary file 1 — Figure S1. (A) μCT‐derived trabecular separation (Tb.Sp) and bone mineral content (Tb.BMC) in the distal femoral metaphysis among mice receiving 25 mg/kg of antibody at different relative proportions of Scl‐Ab and Dkk1‐mAb, at 16 weeks of age. (B) μCT‐derived trabecular separation (Tb.Sp) and bone mineral content (Tb.BMC) in the 5th lumbar vertebra among all treatment groups, at 16 weeks of age. *p < 0.05 vs. vehicle; #p < 0.05 vs. Scl‐mAb alone; n = 6–7 mice/group. Figure S2. Quantification of anabolic action on the (A) endocortical (Ec) and (B) periosteal (Ps) surfaces, measured using labels administered at the start (9 weeks) and near the end (14 weeks) of the antibody treatment period, and presented as the mineralizing surface per unit bone surface (MS/BS) and the mineral apposition rate (MAR). Mice received 25 mg/kg of antibody at different relative proportions of Scl‐Ab and Dkk1‐mAb, *p < 0.05 vs. vehicle; #p < 0.05 vs. Scl‐mAb alone; n = 6–7 mice/group. Figure S3. μCT‐derived trabecular separation (Tb.Sp) and bone mineral content (Tb.BMC) in (A) the distal femoral metaphysis and (B) the 5th lumbar vertebra among mice receiving different doses of 3:1 Scl‐mAb/Dkk1‐mAb, compared to vehicle control and Scl‐mAb alone (at 25 mg/kg). *p < 0.05 vs. vehicle; #p < 0.05 vs. Scl‐mAb alone; n = 5–6 mice/group. Figure S4. Quantification of anabolic action on the (A) endocortical (Ec) and (B) periosteal (Ps) surfaces, measured using labels administered at the start (9 weeks) and near the end (14 weeks) of the antibody treatment period, and presented as the mineralizing surface per unit bone surface (MS/BS) and the mineral apposition rate (MAR). Mice received different doses of 3:1 Scl‐mAb/Dkk1‐mAb (blue rectangles), 25 mg/kg of Scl‐mAb alone (red rectangles), or vehicle control (gray rectangles). *p < 0.05 vs. vehicle; #p < 0.05 vs. Scl‐mAb alone; n = 5–6 mice/group. [file JBM4-5-e10462-s001.pdf]
